# Shear-Thickening Fluid Using Oxygen-Plasma-Modified Multi-Walled Carbon Nanotubes to Improve the Quasi-Static Stab Resistance of Kevlar Fabrics

**DOI:** 10.3390/polym10121356

**Published:** 2018-12-06

**Authors:** Danyang Li, Rui Wang, Xing Liu, Shu Fang, Yanli Sun

**Affiliations:** 1 School of Textile Science and Engineering, Tianjin Polytechnic University, No. 399 Bin Shui Xi Road, Xi Qing District, Tianjin 300387, China; strawberry4173@163.com (D.L.); liuxing@tjpu.edu.cn (X.L.); fairyfangshu@gmail.com (S.F.); yls198959@163.com (Y.S.); 2 Key Laboratory of Advanced Textile Composites, Ministry of Education, Tianjin Polytechnic University, Tianjin 300387, China

**Keywords:** O_2_-plasma modified MWNTs, shear-thickening fluid, quasi-static stab resistance, body armors

## Abstract

The excellent mechanical property and light weight of protective materials are vital for practical application in body armor. In this study, O_2_-plasma-modified multi-walled carbon nanotubes (M-MWNTs) were introduced into shear-thickening fluid (STF)-impregnated Kevlar woven fabrics to increase the quasi-static stab resistance and decrease the composite weight. The rheological test showed that the addition of 0.06 wt. % M-MWNT caused a marked increase in the peak viscosity from 1563 to 3417 pa·s and a decrease in the critical shear rate from 14.68 s^−1^ to 2.53 s^−1^. The storage modulus (G′) and loss modulus (G″) showed a higher degree of abrupt increase with the increase of shear stress. The yarn pull-out test showed that the yarn friction of M-MWNT/STF/Kevlar fabrics was far superior to the original fabrics. Importantly, under similar areal density, the M-MWNT/STF/Kevlar fabrics could resist 1261.4 N quasi-static stab force and absorb 41.3 J energy, which were much higher than neat Kevlar fabrics. The results of this research indicated that quasi-static stab resistance was improved by M-MWNTs, which was attributed to the excellent shear-thickening effect and the high yarn friction. Therefore, M-MWNT/STF/Kevlar fabrics have a broad prospect in the fields of body protection.

## 1. Introduction

Humans have been using various forms of body armor to protect themselves from dangerous weapons in the wake of rising terrorism and regional conflicts globally [1]. Moreover, in many countries, the restrictions on gun ownership have led to increasing knife attacks [2]. Therefore, an increasing number of researchers are focusing on improving the stab resistance of materials [3,4]. High-performance woven fabrics such as aramid (e.g., Kevlar^®^ and Twaron^®^), and ultra-high-molecular-weight polyethylene (e.g., Spectra^®^ and Dyneema^®^) are widely used in the field of stab-resistant body armor. These are high-strength, high-modulus, high-tenacity fabrics, which can provide protection for users [5]. However, the traditional fabric armor requires multiple layers (approximately 30–50 layers) to ensure the protection level, which is inflexible, heavy, and restricts the agility of the wearer [6]. In recent years, shear-thickening fluid (STF) has been combined with these high-performance fabrics to develop soft body armor, which exhibits good protection performance and overcomes action limitation [7,8]. 

STF is a specific type of non-Newtonian fluid whose viscosity increases dramatically once the external shear rate or applied loading is beyond a critical value [9,10]. This smart fluid has a reversibility, which transforms from a liquid to solid-like state when subjected to high-speed impact, and returns to the initial liquid state when the impact stress disappears [11]. Wagner et al. [12] firstly impregnated the STF with aramid Kevlar^®^ woven fabric to develop the STF/Kevlar soft armor, and found that four layers STF/Kevlar composites absorbed more energy than 14 layers of neat Kevlar fabrics. Thus, various research has been done to investigate the shear-thickening effects and stab resistance of STF-treated fabrics. A series of influence factors, such as particle concentration [13], particle size [14], particle hardness [15], dispersing medium [16], and weave structure [17], have been systematically evaluated. To further enhance the stab resistance of fabrics, nanofillers were explored to promote the effectiveness of STF [5,18,19,20]. Some previous work is summarized in Table 1. Aranya Ghosh et al. [19] investigated the influence of cellulose nanofibers (CNFs) on the rheological characteristics of silica-based STF. According to their study, just 0.2 wt. % CNFs caused a marked increase in the peak viscosity, and a shear-thickening mechanism was proposed. Animesh Laha et al. [20] found that the addition of 0.05 wt. % halloysite nanotubes (HNT) significantly enhanced the peak viscosity and decreased the critical shear rate of STF. Moreover, the addition of Hal nanotubes increased the impact energy absorption by between 40% and 60% towards different types of Kevlar fabrics. 

MWNT is an ideal nano-additive to reinforce and functionalize traditional materials due to its outstanding physicochemical properties of large aspect ratio, high flexibility and strength, and low density [21,22]. However, the strong van der Waals interactions and the hydrophobicity of MWNTs lead to agglomeration in STF [23]. To overcome these problems, surface-modified MWNTs, as with plasma treatment, has been proved to be effective. Compared with chemical methods, the plasma treatment has many advantages, such as non-polluting properties, shorter reaction time, and so on [24,25]. However, a study on the effect of O_2_-plasma-modified MWNTs on the rheological behavior of concentrated colloidal dispersions and quasi-static stab resistance performance is still quite limited. In this work, we present a novel composite based on Kevlar and STF-containing O_2_-plasma-modified MWNTs. The M-MWNT/STF were fabricated through mechanical stirring, fumed silica and O_2_-plasma-MWNTs as a dispersing phase and polyethylene glycol as a dispersing medium, aiming to improve the rheological properties of the STF and the stab resistance of the fabrics. A series of experiments, including Raman spectroscopy analysis, rheology test, yarn pull-out test, and quasi-static stab test, have been carried out to prove the enhancement effect. The enhancing mechanism of the quasi-static stab resistance has been also discussed.

## 2. Experimental

### 2.1. Materials

**Figure 1:**
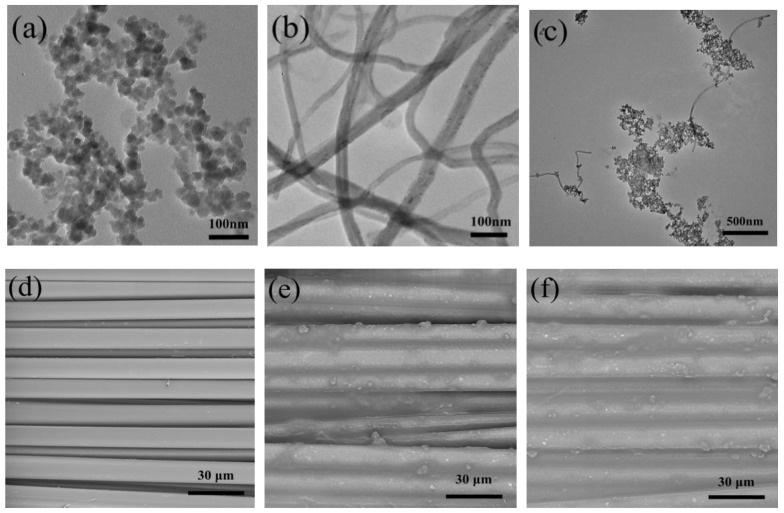
TEM images of (**a**) fumed silica nanoparticles, (**b**) O_2_-plasma treatment MWNTs and (**c**) fumed silica nanoparticles and M-MWNTs, SEM images of (**d**) neat Kevlar fabric, (**e**) STF/Kevlar fabric, and (**f**) M-MWNT/STF/Kevlar fabric.

MWNTs with the length and diameter of 1–30 μm and 10–50 nm were obtained from Nanjing XF Nanomaterial Science and Technology Co., Ltd. (Nanjing, China). Fumed silica nanoparticles (AEROSIL200) with a primary particle size 12 nm and specific surface area of 200 m^2^/g were supplied by Degussa (Frankfurt, Germany). Figure 1a shows the transmission electron microscope of fumed silica nanoparticles. Polyethylene glycol (Analytical Reagent) with an average molecular weight of 200 g mol^−1^ was purchased from Tianjin Kemiou Chemical Reagent Co., Ltd. (Tianjin, China). Ethanol absolute (Analytical Reagent) was offered by Tianjin Fengchuan Chemical Reagent Science and Technology Co., Ltd. (Tianjin, China). The Kevlar fabrics used in this study were purchased from Dupont Company (Wilmington, DE, USA). The specifications of Kevlar fabrics are shown in Table 2.

### 2.2. Preparation

#### 2.2.1. Preparation of the O_2_-Plasma-Modified MWNTs

**Figure 2:**
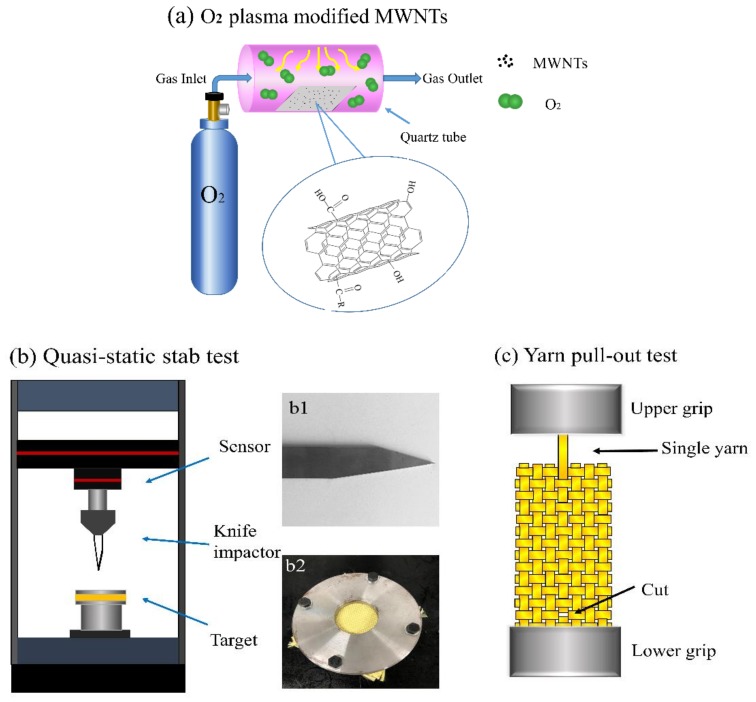
Schematic illustration of experimental equipment. (**a**) O_2_ plasma modified MWNTs. (**b**) Quasi-static stab test, the knife blade P1 (b1) and matched to the fabrics clamper (b2), (**c**) Yarn pull-out test.

An radio frequency (RF) plasma source was used for functionalizing the MWNTs. Figure 2a shows a schematic diagram of how MWNTs are modified in the plasma equipment. The oxygen plasma treatment conditions for the MWNTs were as follows: The MWNTs were placed into a quartz tube right below the plasma with an oxygen flow rate of 100 sccm, a voltage of 220 V, an operating pressure of 15 Pa, and a power of 150 W and elapsed time for 5min. Figure 1b shows the micrographs of the M-MWNTs.

#### 2.2.2. Preparation of STF 

STF was prepared by gradually adding fumed silica nanoparticles into a PEG-200, stirred by a mechanical mixer until the loading of the silica nanoparticles achieved a desired concentration. To get well-dispersed STF suspensions, a high-speed dispersator (XHF-D) was used at a speed of 8000 rpm to stir the suspensions. The silica loading in the suspension was 25 wt. %, 30 wt. %, and 35 wt. % to investigate the rheological behavior of STF. Due to the high free surface energy of 12 nm silica nanoparticles, 35 wt. % silica loading was the maximum concentration possible. To prepare the STF compounded M-MWNTs, the silica nanoparticles were added to the well-dispersed and sonicated M-MWNT/PEG-200 solution. Because of the better dispersibility of the M-MWNTs in STF, the mass friction 0.02%, 0.04%, 0.06% of M-MWNTs were selected, meanwhile the silica loading of the suspension was 35 wt. %. All the suspensions were placed in a vacuum oven for 1h to remove air bubbles prior to rheological measurement. 

#### 2.2.3. Preparation of STF-Treated Fabric 

First, the STF was diluted with ethanol 1:1 (*v*/*v*) to reduce the surface tension and viscosity. The Kevlar weave fabric was cut into square specimens of 10 cm × 10 cm. Then, each of the fabrics was immersed into the diluted STF for 1 min. To remove excessive fluid, the infiltrated fabrics were padded at 1.5 bar pressure and 2 m/min using a pair of rubber rollers. Finally, the fabrics were put into a hot-air oven at 60 °C for 2 h to evaporate the ethanol. Figure 1c shows the distribution and morphology of STF with 0.06 wt. % M-MWNTs.

### 2.3. Characterizations 

Raman spectroscopy of the pristine and M-MWNTs was measured on a laser Raman spectrometer (XploRA PLUS Horiba, Kyoto, Japan) with the incident laser wavelength of 532 nm.

The morphology of the fumed silica nanoparticles, M-MWNTs, and M-MWNT/STF was characterized with a transmission electron microscope (TEM, Hitachi H 7650, Tokyo, Japan). The surface morphology of neat and STF-treated fabrics (both STF/Kevlar fabrics and M-MWNT/STF/Kevlar fabrics) were characterized by a scanning electron microscope (SEM, Phenom LE, Micromeritics Instrument Corp., Georgia, USA). All the samples were sputter-coated with gold by an ion-sputtering instrument (SBC-12) for 120 s at room temperature. As seen from the SEM images in Figure 1d, the surface of the untreated fiber is clean and smooth and there are large gaps among the fibers. Compared to STF-treated fibers, a large number of SiO_2_ nanoparticles evenly covered the fiber surfaces and filled the gaps among the fibers. 

The rheological properties of STFs were measured using a stress-controlled rheometer (Bohlin CVO, Malvern, UK) with a parallel plate apparatus of 20 mm. The gap between the plates was maintained at 0.3 mm. With the help of the temperature-control device, measurements took place at the fixed temperature of 25 °C. The steady-state rheological behavior was observed under a wide range of shear rates, from 0.03–1000 s^−1^. The viscoelastic behaviors were performed by a stress sweep from 1 to 1000 pa at a constant frequency of 10 Hz.

The quasi-static stab test of the composites was performed using a knife blade P1 at a speed of 508 mm/min referring to ASTM 1342 and Nation Institute of Justice (NIJ) standard-0115.00, as shown in Figure 2b. The sample was clamped by a self-designed hollow cylindrical holder with an outside diameter of 15 cm and an inner diameter of 5 cm. To avoid slippage, a sufficient force was used to clamp the target with four screws. Then a knife impactor was pushed into the center of the target and, during the stab process, load-displacement data was obtained.

Yarn pull-out test was carried out to investigate the effect of STF and M-MWNT/STF on the friction between yarn–yarn. The fabrics were cut into pieces with a size of 95 × 20 mm, in reference to the previous literature [7]. The top of the warp direction yarn was fixed on the movable grip; meanwhile, the lower part of the specimen was mounted in the lower grip. The lower end of the single yarn was intentionally cut to be pulled out without elastic deformation or failure. All the tests were measured using an Instron 5969 machine (Norwood, MA, USA) and the testing speed was 100 mm/min. Figure 2c shows the schematic diagram.

## 3. Result and Discussion

### 3.1. Raman Spectroscopy

**Figure 3:**
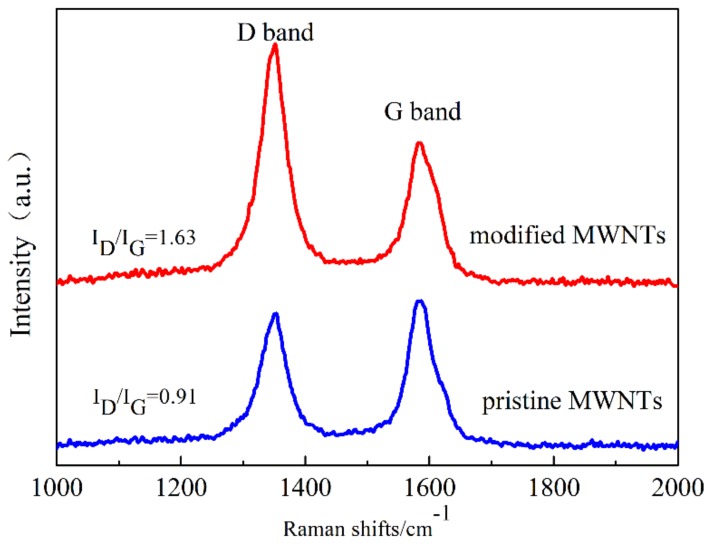
Raman spectra of pristine MWNTs and O_2_-plasma-modified MWNTs.

The *Raman* spectra of pristine MWNTs and O_2_-plasma-modified MWNTs are shown in Figure 3. The characteristic peaks of pristine MWNTs are observed around wavenumber of 1351 cm^−1^ and 1588 cm^−1^ corresponding to the D and G bands, respectively. The M-MWNTs showed an obvious enhancement of the D band and a blueshift of the G band. This is due to an increase number of sp^3^ hybridized bonds and the oxygen-containing functional groups presented on the surface of the MWNTs. The value of intensity ratio of the D band and G band (I_D_/I_G_) is used to characterize the structural defeats and the covalent functionalization [28]. After being modified, the I_D_/I_G_ value of M-MWNTs increases from 0.91 to 1.63 because of the increasing oxygen content. The Raman spectra showed that the oxygen-containing functional groups were grafted onto the MWNTs’ sidewalls and ends, which improved the dispersibility of MWNTs in STF. 

### 3.2. Rheological Behavior of STFs

**Figure 4:**
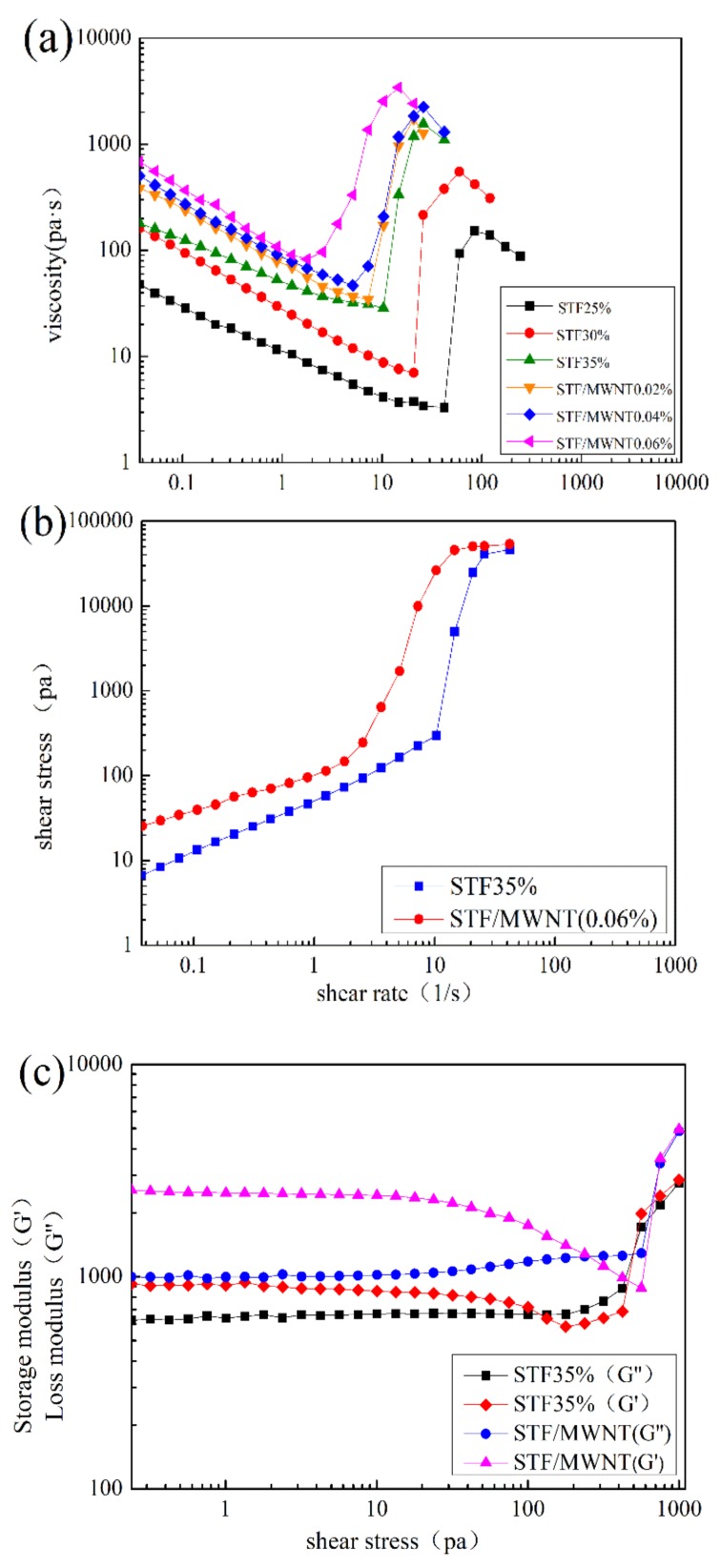
(**a**) Viscosity and (**b**) shear stress versus shear rate; (**c**) Storage (G′) and loss (G″) modulus versus shear stress for 35% STF and 0.06%M-MWNT/STF.

The steady rheological property of STF and M-MWNT/STF is shown in Figure 4a. It was clearly observed that the weight fraction increase of silica nanoparticles caused a decrease in critical shear rate, which triggered the shear thickening and an increase in peak viscosity. The rheological properties and fitting function are summarized in Table 3. Each curve in Figure 4a was fitted from the lowest point to the highest point. According to the fitted function, it could be clearly seen that the STF with 35 wt. % silica nanoparticles had a better rheological property in all the STF samples without M-MWNTs. Hence, the STF with 35 wt. % silica nanoparticles was chosen to investigate the effect of M-MWNTs in the STF. It was also found that the STF with 0.06 wt. % M-MWNTs showed the most outstanding rheological properties in all the STF samples. The addition of 0.06 wt. % M-MWNTs to the STF caused a marked increase in the peak viscosity, from 1563 to 3417 pa·s, and a concomitant decrease in the critical shear rate from14.68 s^−1^ to 2.53 s^−1^. Thus, STF with 35 wt. % silica nanoparticles and STF with 0.06 wt. % M-MWNTs were selected for the further investigation in this work. 

Figure 4b shows the increase of shear stress with shear rate. With the increasing of shear rate, the shear stress increased slowly and followed a sudden enhancement around the critical shear rate. The M-MWNT/STF displayed a higher yield stress in comparison with the original STF. Figure 4c represents storage modulus (G′) and loss modulus (G″) for the two different compositions STFs as a function of applied shear-stress amplitude. At low shear-stress amplitude, both G′ and G″ exhibited a linear behavior, which implied the microstructure of the system remains stable. Meanwhile, the G′ dominating the G″ led to a soft solid-like behavior existing in two kinds of STF. As the shear stress increased, the G′ declined gradually until G′ was lower than G″ and then followed a sharp increase of both G′ and G″. For M-MWNT/STF, the gap between G′ and G″ is larger than that of the original STF and the elastic nature of the whole system is improved. These results can be attributed to the stronger network formed by M-MWNTs and its interaction with SiO_2_ nanoparticles [26]. This is also the reason that the crossover point shifts towards the higher shear stress, and the whole system needs a higher external stress to break the balance. 

**Figure 5:**
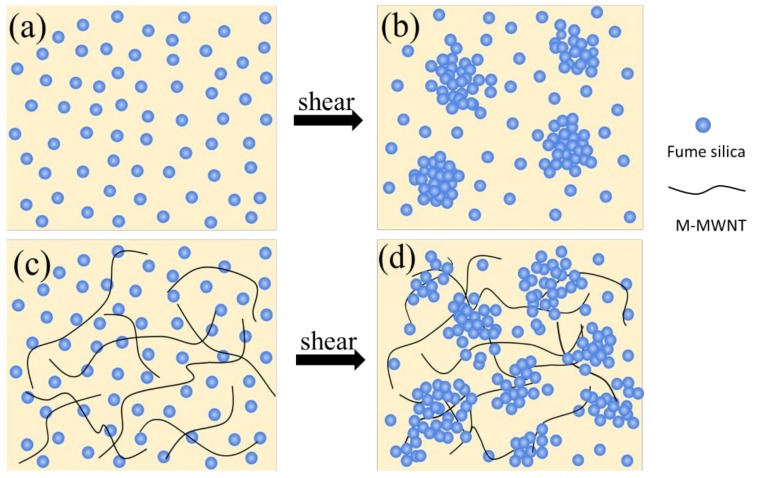
The mechanism of shear-thickening behavior of (**a**) original STF, (**b**) original STF under shear, (**c**) M-MWNT/STF, and (**d**) M-MWNT/STF under shear.

Figure 5 shows the shear-thickening mechanism of the original STF and M-MWNT/STF. The mechanism could be explained by the “hydrocluster” theory, proposed by Bossis and Brady [29,30,31]. With the shear rate increase, hydrodynamic lubrication forces disrupt the stable system resulting in the formation of particle aggregates. Under the hydrodynamic lubrication forces, the particle aggregates collide each other forming hydroclusters, which hinders the flow of fluid. However, the addition of M-MWNTs increases the viscosity of the system and decreases the critical shear rate. The reason could be that there are some oxygen-containing functional groups on the surface of the M-MWNTs, such as hydroxyl groups and carboxyl groups. Thus, these oxygen-containing functions could form hydrogen bonds with silanol groups in the surface of silica, which improves the interaction between the M-MWNTs and silica particles. As a result, the particles travel a shorter distance to form the “hydrocluster” and the shear-thickening occurs at a lower shear rate [19]. Additionally, the MWNTs with a high aspect ratio entangled easily and formed networks, which could enhance the viscosity of the system [27].

### 3.3. Yarn Pull-Out Test Results

**Figure 6:**
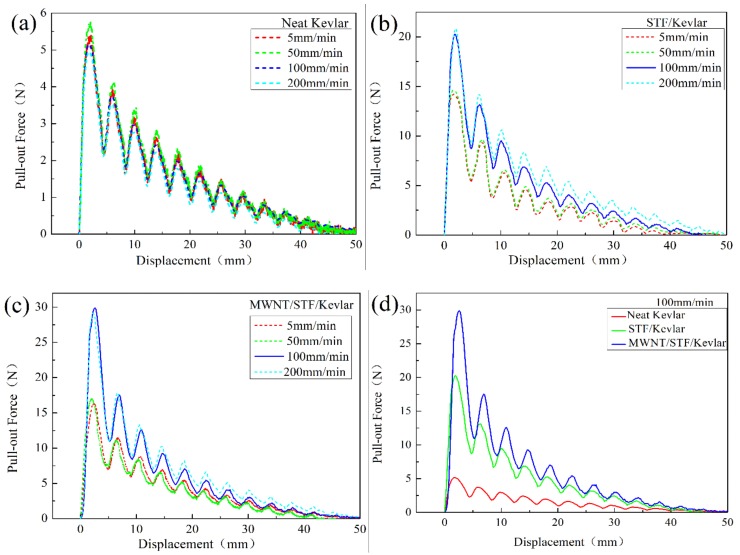
(**a**) Yarn pull-out force versus displacement curves of neat Kevlar. Pull the yarn at a constant speed at 5, 50, 100, 200 mm/min, (**b**) STF/Kevlar, (**c**) MWNT /STF/Kevlar (**d**) and curves of different fabric composite at the speed of 100 mm/min.

The yarn pull-out tests were carried out to investigate the friction between yarn–yarn. As shown in Figure 6a, the pull-out force of neat Kevlar fabrics is almost 5 N even though the pull-out speed varied from low to high values. The reason is that its smooth surface of Kevlar fiber provides poor yarn friction. When the fabrics were impregnated by STF or M-MWNT/STF, the peak pull-out force was always higher than that of neat fabrics no matter the pull-out speed. As shown in Figure 6b,c, at speeds of 5 or 50 mm/min, the peak pull-out force is 14.6 N and 17 N, almost double that of neat fabrics. Moreover, when the speed was 100 or 200 mm/min, the peak pull-out force increases to 20.8 N and 30 N, respectively. It could be concluded that the impregnated fabrics were coated with SiO_2_ nanoparticles, which had a coarse surface resulting in a higher yarn friction. When the pull-out speed exceeds the critical value, the hydroclusters formed by nanoparticles will induce the shear-thickening effect in the dynamic process. The STF covered on the fiber surfaces hinders the mobility of fibers and yarns so that the STF-treated fabrics exhibit an increased pull-out force. A similar behavior of yarn pull-out test has also been reported elsewhere [11,32]. Figure 6d shows that the peak pull-out force of M-MWNT/STF/Kevlar fabric is 30 N, which is increased by 44% compared to that of STF/Kevlar fabrics. This result also indirectly demonstrates that M-MWNTs play an important role in forming hydroclusters, which promote the shear-thickening effect. To prove the relationship between pull-out speed and force, the fitting functions of pull-out force are summarized in Table 4. The function fits all the highest points of each peak in the curve. It could be clearly seen that the pull-out force of neat Kevlar fabrics was hardly dependent on the pull-out speed. Meanwhile, for both STF/Kevlar and M-MWNT/STF/Kevlar fabrics, the pull-out force would apparently increase, once the speed exceeded the critical pull-out speed.

### 3.4. Quasi-Static Stab Test

**Figure 7:**
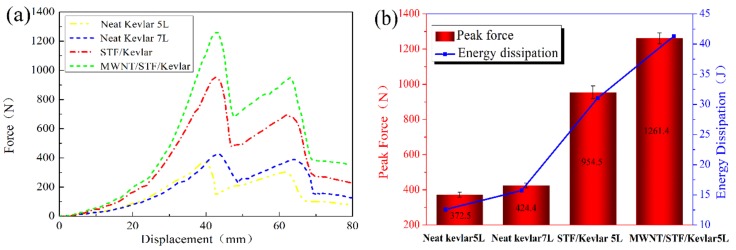
(**a**) Force-displacement curves for quasi-static loading of neat Kevlar fabrics, STF/Kevlar fabrics and M-MWNT/STF/Kevlar fabrics; (**b**) The energy dissipation during the impact process and the peak force.

Table 5 shows the parameters of samples for the quasi-static stab test. Figure 7 shows the force-deformation relationship diagram and quasi-static stab forcing results. The quasi-static stab process of fabrics in Figure 7a could be explained as follows: at the beginning of the stab process, the knife contacts with the targeted fabric. As the stab load increases gradually, the knife begins to pass through the fabric until it penetrates the fabric completely. Meanwhile, the yarns are straightened, tensioned, and cut during this process. As a result, the force increased steeply to the maximum value, oscillated slightly, and dropped suddenly to a low value. Figure 7b shows the maximum quasi-static stab resistance force and the energy dissipation of all the samples. In comparison to the neat Kevlar (372.5 N), the maximum force of M-MWNT/STF/Kevlar fabrics increased dramatically to 1261.4 N with the same five layers (5 L). In addition, the maximum force (1261.4 N) was also 1.3-fold than that of the STF/Kevlar fabrics. The neat Kevlar fabrics (7 L), which has the closest areal density as the STF and M-MWNT/STF Kevlar fabrics, is constructed to explore the influence of areal density on stab resistance. It can be seen that the maximum force for neat Kevlar fabrics (7 L) is 424.4 N, only suffering 13.92% more load and absorbing 25.1% more energy than the neat Kevlar fabrics (5 L). Hence, it could be inferred that a better quasi-static stab resistance of STF-treated fabrics (both STF/Kevlar and M-MWNT/STF/Kevlar) could be originated from the shear-thickening behavior of STF. With the stab force increasing, the hydrodynamic lubrication forces dominate in the system, resulting in SiO_2_ nanoparticle aggregates. When the stab force exceeds a critical value, hydrodynamic lubrication forces cause collisions among nanoparticle aggregates, resulting in hydroclusters. Consequently, the viscosity of the system increased drastically leading to soft fabrics becoming rigid. Importantly, after O_2_ plasma treatment, the oxygen-containing functional groups grafted on the MWNTs interacts with silica, which induces the quick formation of large hydroclusters. Hence, the M-MWNT/STF/Kevlar fabrics could resist more force and dissipate more energy than the STF/Kevlar fabrics. 

**Figure 8:**
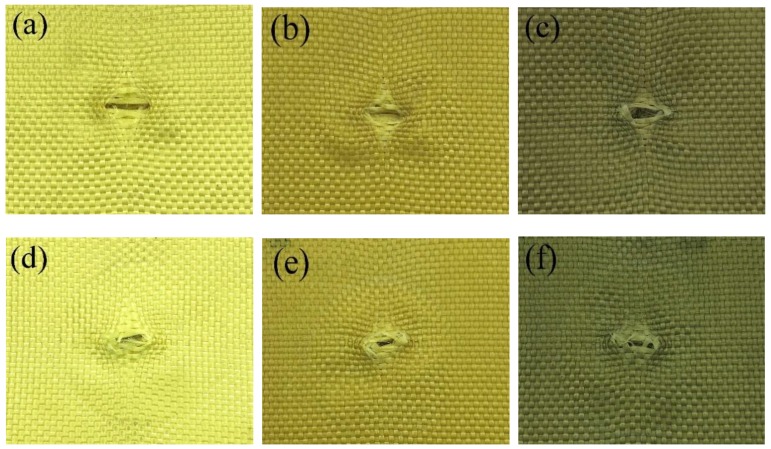
Front and rear view of samples after the quasi-static tests: (**a**,**d**) neat Kevlar fabric, (**b**,**e**) STF/Kevlar fabric, (**c**,**f**) M-MWNT/STF/Kevlar fabric.

Another reason could be that STF on the fabrics restricts the mobility of yarns and fibers. Figure 8 shows the damage of neat and STF-treated fabrics after quasi-static stab tests against a knife. As shown in Figure 8a,d, due to yarn slippage, the knife can pass through neat fabrics easily, which causes a large-ranging windowing effect. In contrast, the dominant failure mode of STF-treated fabrics was yarn rupture. The local yarns are cut cleanly and sharply, and a small-ranging windowing effect can be seen in Figure 8b,c. In consideration of the failure mode, the STF-treated fabrics have a high friction between yarn–yarn, which limits the motion of yarns. Thus, more fabric yarns around the target point could participate in load-bearing until yarn breakage [33]. 

Based on the above analysis, the working mechanism of M-MWNT/STF-enhanced fabric stab resistance is explained as follows. First, the high degree of shear-thickening effect of M-MWNT/STF causes an increase in viscosity, which could dissipate part of energy. Secondly, the yarn friction also contributes significantly in energy absorption during the stab process. In general, when the knife stabbed neat fabrics, the strength and friction of the yarns are too small to resist the shearing and separating force, which causes fabric windowing forming easily. Meanwhile, the STF-assisted fibers maintain a stable arrangement and increase the strength of yarns. As a result, the friction of knife–fibers and fiber–fiber increased significantly. Furthermore, due to the stronger shear-thickening effect, the friction of M-MWNT/STF-treated yarns is higher than that of STF/Kevlar fabrics. It could be concluded that adding M-MWNTs to STF is an effective way to enhance the quasi-static stab resistance of fabrics through improving the shear-thickening behavior of STF and the friction between yarn–penetrator and yarn–yarn.

## 4. Conclusions

In this study, a smart and flexible composite with shear-thickening effect was developed by integrating O_2_-plasma-modified MWNTs hybrid-STF and Kevlar fabrics. Raman spectra showed that the oxygen-containing functional groups were successfully grafted onto the MWNTs’ sidewalls and ends by O_2_-plasma treatment. Rheological testing indicated that the M-MWNT hybrid-STF possessed an excellent shear-thickening behavior, where the critical shear rate decreased from 14.68 s^−1^ to 2.53 s^−1^ and the maximum viscosity increased from 1563 to 3417 pa·s. The dynamic oscillatory test showed both G′ and G″ were enhanced by adding M-MWNTs. At the critical shear stress, G′ and G″ underwent an abrupt increase. Additionally, the peak pull-out force of M-MWNT/STF/Kevlar fabrics was 30 N, which increased 5.8-fold compared to that of the neat Kevlar fabrics. In addition, the high yarn friction limited the yarn slippage, which made more yarn around the target dissipate more impact force. Moreover, for the M-MWNT/STF/Kevlar fabrics, the maximum resistant stab force was 1261.3 N, which was rather higher than the neat and STF/Kevlar fabrics with the same layers. In summary, lightweight, flexible, stress-responsive smart materials significantly enhanced the quasi-static stab resistance of fabrics, which offers a bright prospect in the fields of soft body armor, damping devices, and energy absorption.

## Figures and Tables

**Table 1:** Rheological properties comparison of nanofillers added in STF.

Filler Type	Additive Amount (wt%)	SiO_2_ Dia	SiO_2_ (wt. %)	Dispersing Medium	γ_c_ (s^−1^)	η_max_ (pa·s)	Ref.
MWNTs	0.8	12 nm	44	PEG-200	196	77.7	[5]
MWNTs	0.4	2 μm	64	EG	3.5	13.4	[26]
CNTs + GNs	2 + 1	650 nm	75	PEG-200	~0.24	~2302	[27]
GNs	0.8	12 nm	20	PEG-200	~4.2	~105	[18]
CNFs	0.2	500 nm	65	PEG-200	16.6	139	[19]
HNTs	0.05	100 nm	65	PEG-200	40	550	[20]
M-MWNTs	0.06	12 nm	35	PEG-200	2.53	3417	This work

**Table 2:** The properties of Kevlar fabrics.

Fabric Structure Parameters	Value
Weave	Plain
Areal density (g/m^2^)	420
Fabric thickness (mm)	0.56
Warp density (ends/cm)	6
Weft density (ends/cm)	6

**Table 3:** Rheological properties and fitting functions of STF and M-MWNT/STF.

Sample	SiO_2_ wt. %	M-MWNT wt. %	γ_c_^1^ s^−1^	η_max_^2^ pa·s	Fitting Function	R^2^
STF	25	0	59.89	152.3	$y=-0.0642x^{2}+11.629x-372.7$	0.9989
	30	0	29.64	215.2	$y=-0.2295x^{2}+32.174x-555.68$	0.9962
	35	0	14.68	1563	$y=-0.6589x^{2}+127.69x-1271.4$	0.9789
M-MWNT/STF	35	0.02	10.33	1728	$y=1.7205x^{2}+74.67x-723.45$	0.9805
	35	0.04	7.27	2243	$y=1.7208x^{2}+82.803x-503.56$	0.9723
	35	0.06	2.54	3417	$y=12.659x^{2}+122.83x-323.57$	0.9795

^1^ γ_c_ means critical shear rate. ^2^ η_max_ means maximum viscosity.

**Table 4:** The fitting functions of pull-out force.

Sample	V (mm/min)	Fitting Function	R^2^
Neat fabric	50	$y=0.0061x^{2}-0.3243x+5.8987$	0.9895
	100	$y=0.0058x^{2}-0.3097x+5.5456$	0.992
STF/Kevlar	50	$y=0.00232x^{2}-1.118x+16.216$	0.9962
	100	$y=0.0338x^{2}-1.6049x+22.639$	0.9915
M-MWNT/STF/Kevlar	50	$y=0.0294x^{2}-1.3336x+19.334$	0.9897
	100	$y=0.04x^{2}-2.4372x+35.568$	0.989

**Table 5:** The parameters of stab test samples of neat fabrics, STF/Kevlar fabrics and M-MWNT/STF/Kevlar fabrics.

Samples	Number of Layers	Thickness (mm)	Areal Density (g/m^2^)
Neat fabric	5	2.79	2100
Neat fabric	7	3.92	2940
STF/Kevlar	5	3.12	2821.5
M-MWNT/STF/Kevlar	5	3.19	2788.7
